# Knockdown of the Fat Mass and Obesity Gene Disrupts Cellular Energy Balance in a Cell-Type Specific Manner

**DOI:** 10.1371/journal.pone.0038444

**Published:** 2012-06-04

**Authors:** Ryan T. Pitman, Jason T. Fong, Penny Billman, Neelu Puri

**Affiliations:** Department of Biomedical Sciences, University of Illinois College of Medicine, Rockford, Illinois, United States of America; Montreal Diabetes Research Center, Canada

## Abstract

Recent studies suggest that FTO variants strongly correlate with obesity and mainly influence energy intake with little effect on the basal metabolic rate. We suggest that FTO influences eating behavior by modulating intracellular energy levels and downstream signaling mechanisms which control energy intake and metabolism. Since FTO plays a particularly important role in adipocytes and in hypothalamic neurons, SH-SY5Y neuronal cells and 3T3-L1 adipocytes were used to understand how siRNA mediated knockdown of FTO expression alters cellular energy homeostasis. Cellular energy status was evaluated by measuring ATP levels using a luminescence assay and uptake of fluorescent glucose. FTO siRNA in SH-SY5Y cells mediated mRNA knockdown (−82%), increased ATP concentrations by up to 46% (P = 0.013) compared to controls, and decreased phosphorylation of AMPk and Akt in SH-SY5Y by −52% and −46% respectively as seen by immunoblotting. In contrast, FTO siRNA in 3T3-L1 cells decreased ATP concentration by −93% (p<0.0005), and increased AMPk and Akt phosphorylation by 204% and 70%, respectively suggesting that FTO mediates control of energy levels in a cell-type specific manner. Furthermore, glucose uptake was decreased in both SH-SY5Y (−51% p = 0.015) and 3T3-L1 cells (−30%, p = 0.0002). We also show that FTO knockdown decreases NPY mRNA expression in SH-SY5Y cells (−21%) through upregulation of pSTAT3 (118%). These results provide important evidence that FTO-variant linked obesity may be associated with altered metabolic functions through activation of downstream metabolic mediators including AMPk.

## Introduction

The Fat Mass and Obesity Associated gene (FTO) associates with body mass index (BMI), waist circumference, type II diabetes, and other obesity related traits [1] in individuals that are homozygous for the FTO risk allele [2], 16% in most world-wide populations, establishing itself as the focus of intense research. Substantial evidence suggests that the primary physiological effect of variations in the FTO locus in humans is increased dietary intake without significant effects on physical activity or metabolic rates [3], [4]. Murine studies of FTO function demonstrate FTO’s strong influence over energy intake but are complicated by metabolic disturbances including alterations in basal metabolic rate and overall energy expenditure not apparent in human studies [4], [5], [6], [7]. Consequently, current studies have not determined a potential mechanism which results in increased energy intake associated with FTO gene variants in humans.

Tissue-specific gene expression profiling suggests that FTO is highly expressed in the hypothalamus [8], an area of the brain strongly linked to eating behavior. FTO is also expressed at a lower level and maintains functionality in adipose and other tissues [9], [10]. Further studies suggest that FTO functions as both a sensor of energy status [9], [11] and a modulator of metabolic processes [12], and its cellular function may be cell-type specific, since fasting increases FTO expression in white adipose tissue [9] and hypothalamic neurons [11] but decreases FTO expression in brown adipose tissue [9].

Intracellular ATP levels affect cellular function and are tightly controlled through a variety of sensors and signaling pathways [13]. Additionally, intracellular energy status regulated by AMP Kinase plays an important role in controlling organismal energy intake and energy metabolism by hypothalamic neurons [14]. AMPk (5′-Adenosine Monophosphate Activated Protein Kinase) appears to be the primary sensor of intracellular ATP levels in the hypothalamus, as it is phosphorylated in the presence of increased AMP/ATP ratio by the tyrosine kinase LKB1 [15], and is activated by starvation and inhibited by leptin [14]. Further, hypothalamic AMPk controls neuropeptide expression, thereby controlling eating behavior [16], and may control adipocyte function as well [13].

Our results suggest that downregulation of FTO by siRNA in both neuronal and adipocyte cells in culture causes a cell-type specific alteration in cellular ATP levels with a corresponding alteration in AMPk phosphorylation. We thus hypothesize that if FTO’s metabolic functions control AMPk in the hypothalamus, then AMPk may be the primary driver of the systemic metabolic effects seen in human and murine studies of FTO function.

## Results

### Validation of siRNA-induced Knockdown of FTO Gene Expression

**Figure 1:**
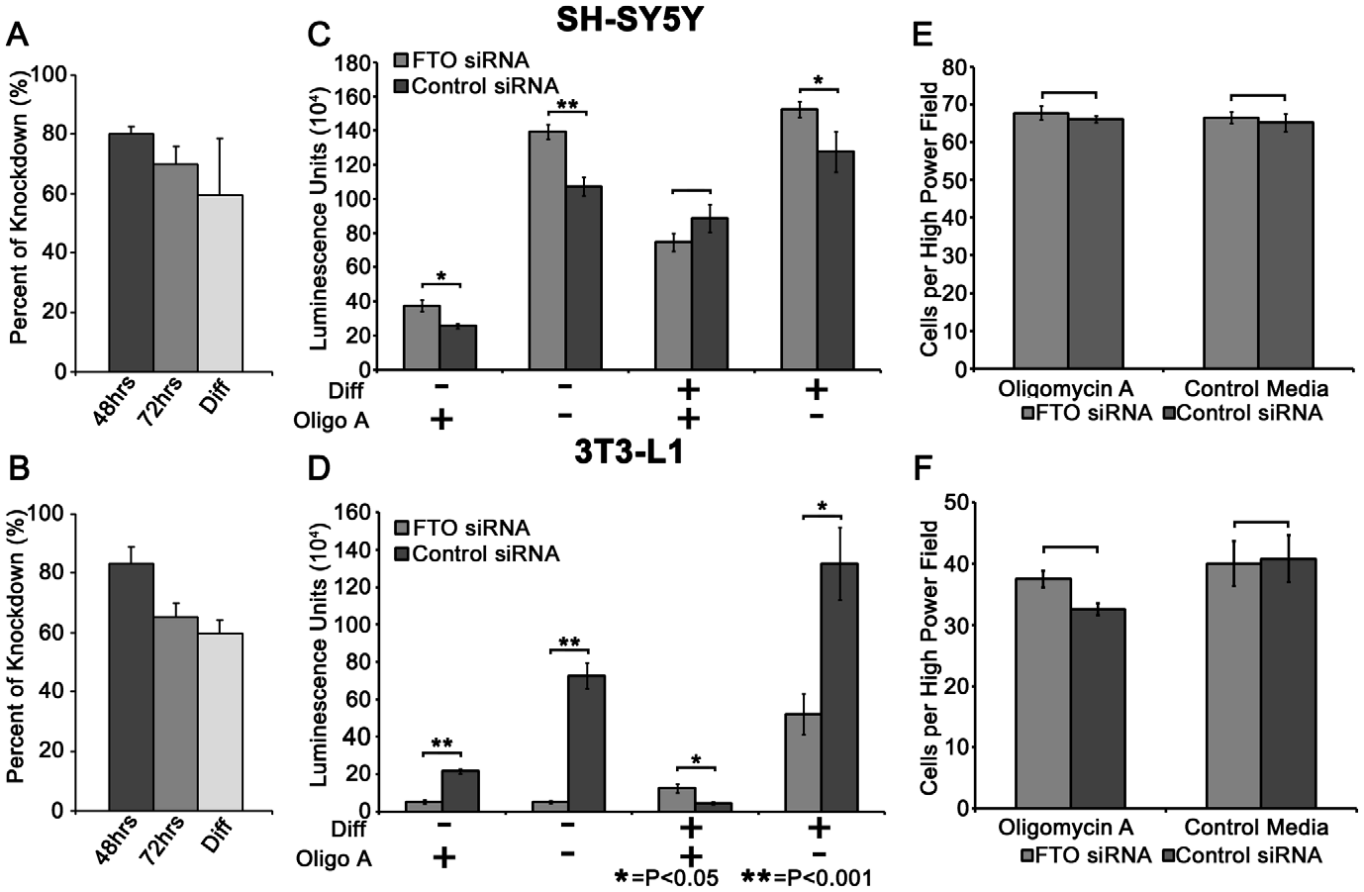
Downregulation of FTO mRNA expression in SH-SY5Y cells and its effects on ATP concentration. (**A**) SH-SY5Y cells were transfected with siRNA for 24 hours and then incubated for a total of 48 hrs (−81.8%) or 72 hrs (−69.8%) post-transfection. Additionally, SH-SY5Y cells were transfected with siRNA for 48 hrs and then differentiated for 1 week (−59.3%). GAPD mRNA was used as a positive internal control. (**B**) 3T3-L1 cells were transfected as described above and FTO expression was decreased by −80.3% at 48 hrs post-transfection, 65.3% at 72 hrs post-transfection, and 59.6% after 48 hrs of transfection and 1 week of differentiation. (**C, D**) FTO knockdown modulates ATP levels in SH-SY5Y and 3T3-L1 cells. Cells were transfected with siRNA and then incubated for 48 hrs post-transfection or differentiated for 1 week. (**C**) ATP levels were increased in naïve SH-SY5Y cells treated with glucose free DMEM only (46%, p = 0.013), oligomycin (30%, p<0.0001), and differentiated SH-SY5Y cells treated with only glucose-free DMEM (16% p = 0.05), but decreased in differentiated SH-SY5Y cells treated with oligomycin (−16%, p = 0.19). (**D**) ATP levels were decreased in naïve 3T3-L1 cells treated with glucose free DMEM only (−93%, p = 0.0005), oligomycin (−76%, p = 0.00002), and differentiated 3T3-L1 cells treated with only glucose-free DMEM (−54% p = 0.0068), yet increased in differentiated 3T3-L1 cells treated with oligomycin (179%, p = 0.026). Values indicate percent of control. ATP results are average of 2 independent experiments performed in at least quadruplicate and analyzed by one-way ANOVA with post-hoc analysis with α at 0.05. (**E, F**) FTO knockdown does alter cell viability of SH-SY5Y and 3T3-L1 cells. Cells were transfected with siRNA, incubated for 48 hrs and viable cells were collected and counted. (**E**) No difference was found between FTO siRNA treated SH-SY5Y cells and Control siRNA treated cells (p = 0.35). In addition, no difference in the cell viability was detected in FTO siRNA treated SH-SY5Y cells after exposure to oligomycin (p = 0.77). (**F**) There is no difference in FTO siRNA or Control siRNA treated 3T3-L1 cells (p = 0.35) or in Control siRNA treated 3T3-L1 cells treated with oligomycin compared to FTO siRNA treated cells (p = 0.131). Cell viability results are the result of experiments performed in quadruplicate and analyzed by repeated measures ANOVA with α at 0.05.

To confirm appropriate downregulation of FTO in both naïve and differentiated cells, qPCR was performed at several time points after transfection, and one week after induction of differentiation. Naïve (undifferentiated) SH-SY5Y cells treated with siRNA against FTO exhibited a decrease in FTO mRNA expression by −81.8%, and −69.8% at 48 and 72 hours, respectively (Fig 1A). 3T3-L1 cells exhibited −80.3% and −65.3% decrease in FTO mRNA at 48 and 72 hours after transfection, respectively (Fig 1B). Additionally, sustained knockdown of FTO mRNA was validated after 1 week of differentiation in both SH-SY5Y (−59.3%) and 3T3-L1 cells (−59.6%) (Fig 1A and 1B).

### FTO Knockdown Disrupts Cellular Energy Balance in a Cell-type Specific Manner

The FTO gene is known to play an important role in controlling energy balance as previous reports show a strong correlation between FTO expression and organismal energy status, including fat accumulation and energy intake [5]. However, little is known about the relationship between FTO expression and cellular energy balance, and substantial research has shown that alterations in neuronal cellular energy status can have large implications on eating behavior [14]. Thus, to determine if FTO plays a role in maintaining or controlling cellular energy status, we examined the effect of FTO knockdown on ATP levels in both SH-SY5Y neuronal cells and 3T3-L1 adipocytes. To fully evaluate ATP levels after siRNA transfection, naïve or differentiated cells were incubated for 30 minutes in glucose-free media, to inhibit glycolysis, with or without oligoymycin A, a specific inhibitor of ATP synthase, to completely inhibit the production of new ATP.

In naive SH-SY5Y cells, FTO knockdown increased ATP levels in cells treated with oligomycin by 46% (FTO siRNA 37466±3263 Luminescence units (LU) vs Control siRNA 256243±1439 LU; p = 0.013; N = 6) and in cells treated with only glucose-free media by 30% (FTO siRNA 1393330±42889 LU vs Control siRNA 1073143±53304 LU; p<0.0001; N = 6) (Fig 1C). However, in differentiated SH-SY5Y cells, ATP levels increased marginally in cells treated with glucose-free media (16%, FTO siRNA 1524949±45660 LU vs Control siRNA 1276264±117101 LU; p = 0.05; N = 5) (Fig 1C). Interestingly, in differentiated SH-SY5Y cells treated with oligomycin, treatment with FTO siRNA appears to marginally decrease cellular ATP content (−16%, FTO siRNA 746552±52886 LU vs Control siRNA 886431±81662 LU; p = 0.19; N = 5) (Fig 1C). In stark contrast, naïve 3T3-L1 cells treated with FTO-siRNA showed a decrease in ATP concentration by −93% (FTO siRNA 5129±738 LU vs Control siRNA 72768±6759 LU; p = 0.0005; N = 5) and −76% (FTO siRNA 5150±1000 LU vs Control siRNA 21650±1359 LU; p = 0.00002; N = 5) in cells treated with glucose-free media or oligomycin, respectively (Fig 1D). However, differentiated 3T3-L1 cells displayed a decrease in ATP levels only in cells treated with glucose-free media (−54%, FTO siRNA 52323±10932 LU vs Control siRNA 132734±19360 LU; p = 0.0068; N = 6), while differentiated 3T3-L1 cells treated with oligomycin displayed an increase in ATP levels as compared to controls (179%, FTO siRNA 12489±2187 LU vs Control siRNA 4467±790 LU; p = 0.026; N = 4) (Fig 1D). These results suggest that a decrease in FTO gene expression may alter cellular ATP levels and provides additional evidence that FTO’s contribution to obesity is cell-type specific.

### Cellular Proliferation is Unaffected by Exposure to FTO siRNA

To study the effect of FTO siRNA treatment on cellular proliferation in SH-SY5Y and 3T3-L1 cells, we plated and treated cells as described below and subsequently trypsinized and counted viable cells. FTO siRNA did not cause a change in cell viability in either SH-SY5Y (FTO siRNA 66.6±1.4 cells/high power field (hpf) vs Control siRNA 65.2±2.3 cells/hpf; p = 0.35; N = 4) (Fig 1E) or 3T3-L1 cells (FTO siRNA 39.9±3.6 cells/hpf vs Control siRNA 40.8±3.8 cells/hpf; p = 0.77; N = 4) (Fig 1F).

To confirm that changes in ATP levels in response to FTO siRNA are not due to differences in cell survival after oligomycin treatment, FTO siRNA and Control siRNA treated SH-SY5Y and 3T3-L1 cells were exposed to oligomycin for 30 minutes, collected and counted. Exposure to oligomycin does not alter cell survival in FTO siRNA treated SH-SY5Y cells (FTO siRNA 67.8±1.8 cells/hpf vs Control siRNA 66.2±0.9 cells/hpf; p = 0.35; N = 4) (Fig 1E) or 3T3-L1 cells (FTO siRNA 37.4±1.4 cells/hpf vs Control siRNA 32.6±0.9 cells/hpf; p = 0.131; N = 4) (Fig 1F) as compared to Control siRNA treated cells. These results suggest that alterations in ATP levels in response to FTO knockdown are not caused by changes in cell viability.

### Effect of siRNA Targeting the FTO Gene on Cellular Signaling and Glucose Uptake

**Figure 2:**
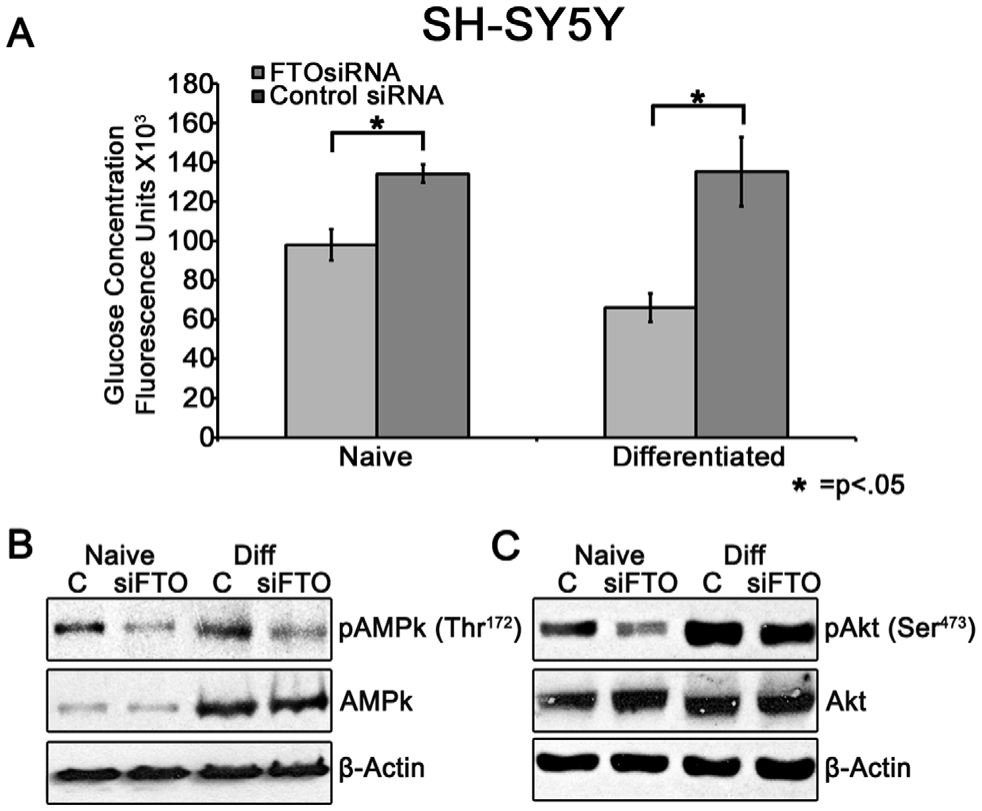
Effect of FTO knockdown on glucose uptake and downstream signaling in SH-SY5Y cells. (**A**) Cells were transfected with siRNA and then incubated 48 hrs post-transfection or differentiated for 1 week. Cells were then exposed to 80 µM 2-NBDG in DMEM media lacking glucose and sodium pyruvate for 5 minutes and glucose-associated fluorescence was measured. Glucose uptake was decreased in both naïve (−27%, p = 0.007) and differentiated (−51% p = 0.015) SH-SY5Y cells. Y-axis represents Arbitrary Fluorescent Units (±SEM) from 2 independent experiments run in quintuplicate and analyzed by one-way ANOVA with post-hoc analysis. (**B**) FTO knockdown decreases AMPk activation. SH-SY5Y cells were transfected with siRNA and then incubated 48 hrs post-transfection or differentiated for 1 week and subsequently immunoblotted. pAMPk (Thr^172^) was decreased in both naïve (−51.8%) and differentiated cells (−52.3%) while total AMPk was unchanged. (**C**) FTO knockdown decreases Akt phosphorylation. pAkt (Ser^473^) was decreased in both naïve (−45.8%) and differentiated cells (−30.0%) while total Akt was unchanged. Blots and densitometry data are from a representative of two similar independent experiments.

**Figure 3:**
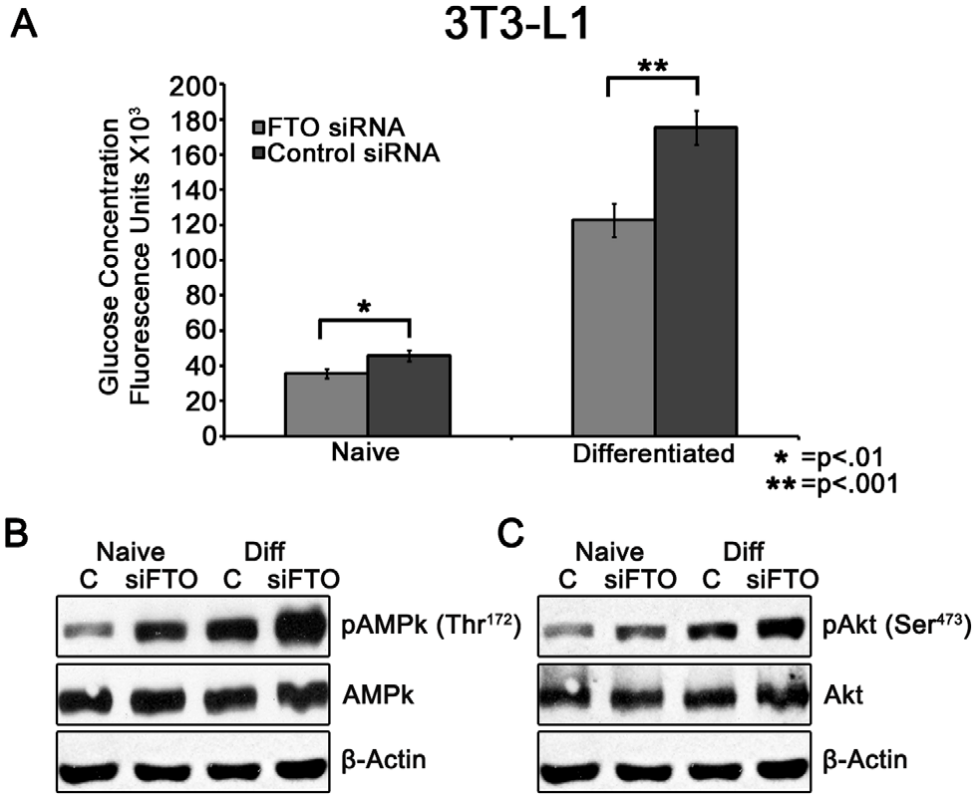
Effect of FTO knockdown on glucose uptake and downstream signaling in 3T3-L1 cells. (**A**) Cells were transfected with siRNA and then incubated 48 hrs post-transfection or differentiated for one week. Cells were then exposed to 80 µM 2-NBDG in DMEM media lacking glucose and sodium pyruvate for 5 minutes and glucose-associated fluorescence was measured. Glucose uptake was decreased in both naïve (−22%, p = 0.017) and differentiated (−30%, p = 0.0002) 3T3-L1 adipocytes. Y-axis represents Arbitrary Fluorescent Units (±SEM) from two independent experiments run in quintuplicate and analyzed by one-way ANOVA with post-hoc analysis. (**B**) FTO knockdown decreases AMPk activation. 3T3-L1 cells were transfected with siRNA and then incubated 48 hrs post-transfection or differentiated for one week and subsequently immunoblotted. pAMPk (Thr^172^) was increased in both naïve (204.4%) and differentiated cells (37.8%) while total AMPk was unchanged. (**C**) FTO knockdown increases Akt phosphorylation. pAkt (Ser^473^) was increased in both naïve (69.9%) and differentiated cells (24.9%) while total Akt was unchanged. Blots and densitometry data from a representative of two similar independent experiments.

After determining the effect of FTO knockdown on cellular ATP levels, we wanted to examine additional metabolic traits modulated by FTO expression. To determine if FTO downregulation affects glucose uptake in SH-SY5Y neuronal cells and 3T3-L1 adipocytes, cells were treated with FTO siRNA and glucose uptake was determined by measuring uptake of fluorescent 2-NBDG. As shown in Figure 2A, glucose uptake into both naïve and differentiated SH-SY5Y cells was significantly decreased by −27% (FTO siRNA 98040±7898 (Fluorescence units (FU) vs Control siRNA 134220±4648 FU; p = 0.007; N = 5) and −51% (FTO siRNA 65952±7281 FU vs Control siRNA 135202±17647 FU; p = 0.015; N = 5), respectively. Additionally, glucose uptake into naïve and differentiated 3T3-L1 adipocytes was decreased by −22% (siRNA 35616±2501 FU vs Control siRNA 45745±3000 FU; p = 0.017; N = 5) and −30% (siRNA 122764±3634 FU vs Control siRNA 174581±3643 FU; p = 0.0002; N = 5), respectively (Fig 3A).

To further explore the metabolic consequences of decreased FTO expression, we immunoblotted for total and phosphorylated AMPK (Thr^172^) (pAMPk) in FTO deficient SH-SY5Y and 3T3-L1 cells. AMPk is an important sensor of cellular energy status [14] that is phosphorylated in the presence of an elevated AMP/ATP ratio by the serine/threonine kinase, LKB1 [17]. AMPk controls multiple cellular metabolic processes including glucose uptake and fatty acid synthesis and may play a role in hypothalamic energy sensing and modulation of eating behavior [14]. As seen in Figure 2B, FTO knockdown decreases AMPk phosphorylation by −51.8% and −52.3% in both naïve and differentiated SH-SY5Y cells, respectively, without a significant alteration in total AMPk levels. However, FTO-deficient 3T3-L1 adipocytes exhibit a significant increase (204% naïve, 38% differentiated) in pAMPK (Thr^172^) without a change in total AMPk (Fig 3B). Since FTO-siRNA does not significantly affect total AMPk protein expression, it is possible that cell-type specific alteration in ATP levels following FTO knockdown are responsible for the cell-type specific alteration in AMPk phosphorylation.

Additionally, Akt, which plays an important role in insulin stimulated glucose uptake [18], is also modulated by FTO knockdown. Akt phosphorylation (Ser^473^) (pAkt) is decreased in both naïve (−46%) and differentiated (−30%) SH-SY5Y cells, without a significant change in total Akt levels (Fig 2C). Inversely, pAkt (Ser^473^) is increased in naïve (70%) and differentiated (25%) 3T3-L1 cells without a significant change in total Akt expression (Fig 3C).

### NPY mRNA Expression is Decreased in FTO-deficient Cells

**Figure 4:**
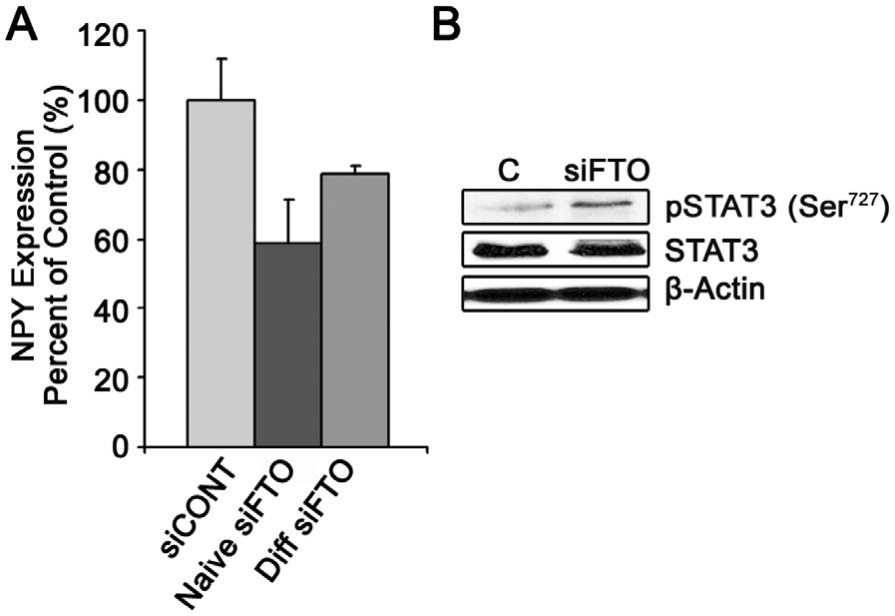
NPY expression in FTO deficient SH-SY5Y cells. (**A**) Cells were transfected with siRNA and then incubated 48 hrs post-transfection or differentiated for 1 week. NPY mRNA levels were then analyzed and were decreased in both naïve (−41%) and differentiated cells (−21%). The Y-axis represents percent of control (±Standard Deviation) of a representative experiment. (**B**) STAT3 activation is decreased in response to FTO knockdown. SH-SY5Y cells were transfected with siRNA and then incubated 48 hrs post-transfection or differentiated for 1 week and subsequently immunoblotted. pSTAT3 (Ser^727^) was increased in differentiated cells (117.5%).

Currently, energy intake is the only obesity-associated trait significantly and repeatedly associated with FTO variants [3], [11], [19], [20], [21] in humans. To determine if FTO downregulation could control the expression of neuropeptides associated with energy intake, we analyzed the mRNA expression of an orexigenic neuropeptide, neuropeptide Y (NPY), that encourages energy intake in the fasted state [22], and an anorexigenic neuropeptide, Proopiomelanocortin (POMC) [23], that discourages energy intake after feeding, in both naïve and differentiated FTO-deficient SH-SY5Y cells. We observed a significant decrease in NPY in both naïve (−41%) and differentiated cells (−21%) (Fig 4A), but no change in POMC mRNA in response to FTO downregulation (data not shown). This corresponds with experiments in mice showing a decrease in NPY expression in FTO knockout mice [7]. Furthermore, we studied the signal transducer and activator of transcription 3 (STAT3), an important regulator of NPY expression [22], that is phosphorylated in the hypothalamus by the leptin receptor to inhibit NPY peptide transcription [22]. In FTO siRNA-treated SH-SY5Y cells, phosphorylated STAT3 (Ser^727^) is upregulated 118% in differentiated cells, while there is no significant change in total STAT3 (Fig 4B).

## Discussion

The study presented in this paper attempts to identify the cellular consequences of FTO downregulation that may account for the influence of FTO variants on obesity. We show that downregulation of FTO gene expression by siRNA significantly modulates cellular concentrations of ATP in both SH-SY5Y neuronal cells and 3T3-L1 adipocytes in a cell-type specific manner. We also demonstrate a cell-type specific response in the phosphorylation of AMPk, an important energy sensor that is involved in controlling both energy expenditure and intake, in response to decreased FTO expression. While these results are preliminary we suggest that our observations may provide the foundation for a mechanism that links FTO expression and the development of human obesity.

Presently, it is unclear how FTO expression may contribute to determining cellular energy status. The FTO gene encodes a 2-oxoglutarate-dependent nucleic acid-demethylase [24] that may function as a transcriptional co-activator through CCAAT/enhancer binding proteins [25]. While previous studies demonstrate that alterations in energy status including fasting and glucose administration [8] may influence FTO gene expression [9], [11], [26], our study is the first to demonstrate a modulation of cellular energy status by changes in FTO expression. We propose that FTO functions as a modulator of cellular energy status exhibited by alteration in ATP concentrations in response to reduced FTO expression.

Our data shows an increase in total ATP concentration in both naive and differentiated FTO-deficient SH-SY5Y cells. In contrast, FTO deficient 3T3-L1 adipocytes exhibit a significant decrease in ATP concentration in both naïve and differentiated cells (Fig 1). Interestingly, these results are reversed in differentiated SH-SY5Y and 3T3-L1 cells treated with oligomycin A, an inhibitor of ATP synthase. These results provide substantial evidence of the involvement of FTO in determining cellular energy status, and show that FTO may influence energy levels in a cell-type specific nature. Unfortunately, the mechanism of this effect is unclear as the observed changes in ATP levels may be caused by alterations in either production or consumption of ATP. Abolishing FTO function significantly alters the expression of the mitochondrial uncoupling protein 2 (Ucp2) [6], and modulation of FTO expression in human myotubes impacts mitochondrial oxidative phosphorylation and alters the expression of mitochondrial genes including ATP5B, UQCR and SOD2 [26]. These combined results suggest the presence of a link between FTO expression and mitochondrial function. However, a link between FTO expression and alterations in ATP consumption must also be explored.

As seen in Figures 2 and 3, naïve SH-SY5Y and 3T3-L1 cells deficient in FTO mRNA display a significant decrease in glucose uptake (−27%, −22%, respectively), with a larger decrease in glucose uptake occurring in differentiated cells (−51%, −30%, respectively). These results corroborate previous evidence that FTO expression modulates glucose metabolism [9], [12], [27]. However, no study has identified a correlation between glucose uptake and FTO gene expression and the mechanism of this effect is unclear. It is possible that the modulation of pAMPk by changes in ATP levels is responsible for the decrease in glucose uptake seen in both SH-SY5Y and 3T3-L1 adipocytes. AMPk phosphorylation decreases glucose uptake in 3T3-L1 adipocytes [28], yet increases glucose uptake in skeletal muscle, neurons, and other tissues [28], [29]. Thus, we suggest the opposing effects of FTO knockdown on AMPk phosphorylation may lead to the decrease in glucose uptake demonstrated in 3T3-L1 and SH-SY5Y cells as seen in Figure 2 and 3. However, the role of AMPk in decreased glucose uptake seen in FTO deficient cells is not clear and requires further experimentation. Additionally, GLUT1, the primary glucose transporter of basal glucose uptake in SH-SY5Y cells [15], [30], is allosterically inhibited by ATP [31], suggesting that increased ATP levels in FTO-deficient SH-SY5Y cells may also potentially contribute to decreased glucose intake. While the mechanism of decreased glucose uptake in response to FTO knockdown is yet to be determined, these results may provide insight into the correlation between FTO variants and altered glucose metabolism demonstrated in previous studies [9], [12], [27].

At present energy intake is the only obesity-associated trait significantly and repeatedly associated with FTO variants [3], [11], [19], [20], [21] in humans. Energy intake is tightly linked to hypothalamic energy balance [32], [33] and is mediated to a significant extent through NPY [14], [22], an orexigenic neuropeptide associated with increased energy intake in fasted mice [16]. We show in this study that FTO downregulation in SH-SY5Y neuronal cells leads to a significant decrease in NPY expression (Fig 4). The observed decrease in NPY mRNA may be modulated by both a decrease in pAMPk [16], previously shown to activate NPY neurons through Ca^2+^ influx [34], and an observed increase in pSTAT3 [14], [22] (Fig 4). While POMC is also modulated by AMPk and STAT3 [23], we observed no significant change in POMC mRNA levels. This data is supported by a decrease in NPY expression observed in FTO knockout mice [7] and an increase in NPY expression observed in FTO knock-in mice [5], and it provides a possible link between modulations in FTO expression and alterations in neuropeptides involved in energy intake.

The conclusions drawn in this study are based upon in vitro experiments performed on immortalized cells and thus, their impact on in vivo feeding behavior are only suggestive/speculative. We encourage further research to confirm a connection between FTO expression and energy intake. However, it is interesting to note that our findings largely support previous findings in which non-functional FTO mutations resulted in decreased food intake and decreased accumulation of fat mass [6], and FTO overexpression resulted in increase in fat accumulation and energy intake in mice [5]. However, Tung et al. demonstrate decreased energy intake in response to stereotactic hypothalamic overexpression of FTO in rats, in stark contrast to the data presented here and demonstrated earlier by others [35]. These results, although very preliminary, coupled with previous findings that attribute variants in the FTO gene to increased food intake [3], [19] and increased FTO mRNA translation [36] suggest that alterations in the transcriptional status in FTO may contribute to obesity by disrupting neuronal cellular energy status, and altering the signaling mechanisms that are responsible for controlling energy intake.

While previous studies by Tung et al on FTO have evaluated the effect of modulation of FTO gene expression in the hypothalamus [35] and other tissues [5], [6], [7], no study has attempted to study the effect of alterations in FTO gene expression in hypothalamic neurons or visceral adipocytes in relation to cellular energy balance and downstream signaling cascades. This study was performed in SH-SY5Y neuroblastoma cells and 3T3-L1 adipocytes, cellular models chosen for their convenience and ability to replicate some characteristics of neurons [37] and adipocytes [38] in vivo. Data for both naïve and differentiated SH-SY5Y neuronal cells and 3T3-L1 adipocytes are presented in this paper since previous results suggest that FTO expression may influence differentiation [39] or FTO inactivity may cause premature senescence or decreased cell survival [25]. Thus, we wanted to determine if differentiation modulated the effect of FTO knockdown on metabolic parameters. In all experiments differentiated cells performed in a similar manner as naïve cells, suggesting a lack of impact of differentiation on FTO knockdown mediated alterations in cellular metabolic activities. However, these results are very preliminary and further experiments must be performed that include knockdown and overexpression of FTO mRNA in hypothalamic neurons and visceral adipocytes with corresponding analysis of cellular energy balance and evaluation of the expression of the proteins modulated in this study.

In summary, this study evaluated the effects of siRNA mediated FTO knockdown on ATP levels and downstream signaling in neuronal and adipocyte cell culture. We are the first to show that alterations in FTO gene expression disrupt cellular energy balance in a cell-type specific manner. Additionally, this study is the first to identify the effects of FTO knockdown on AMPk phosphorylation, and we suggest that AMPk may be a primary mediator of FTO’s influence on energy intake and obesity. These findings provide insight into FTO mediated obesity and support the notion that FTO expression is an important driver of the development of obesity.

## Materials and Methods

### Materials

SH-SY5Y neuroblastoma were acquired from American Type Culture Collection (ATCC, Manassas, VA, CRL-2266) and 3T3-L1 adipocytes were obtained from Elizaveta Benevolenskaya (Department of Biochemistry and Molecular Genetics, University of Illinois at Chicago) and cultured according to the specifications described below. DMEM/F12 (1∶1) (Cat No:11320-033), high-glucose DMEM (Cat No:11965-092), Opti-MEM (Cat No:11058-021), glucose-free DMEM (Cat No:11966-025), 100× penicillin/streptomycin solution (Cat No: 15070-063), 0.05% Trypsin/EDTA (Cat No 25300-120), Molecular grade water(Cat No: 10977-015), HBSS (Cat No: 14025-092), PBS (Cat No: 20012-050), and 2-[N-(7-nitrobenz-2-oxa-1,3-diazol-4-yl) amino]-2-deoxy-d-glucose (2-NBDG) (Cat No: N13195) were purchased from Invitrogen (Carlsbad, CA). Fetal bovine sera (Cat No: S11050) and Calf sera (Cat No: S11450) were purchased from Atlanta Biologicals (Lawrenceville, GA). Retinoic acid (Cat No: R2625), Oligomycin A (Cat No: 7531), Dexamethasone (Cat No: D4902), 3-isobutyl-1-methylxanthine (IBMX) (Cat No: I5879) and insulin (Cat No: I0516) were purchased from Sigma (St. Louis, MO).

The following reagents were used for immunoblotting; phospho-protein extraction buffer (Boston Bioproducts, Ashland, MA, Cat No: BP-116P); nitrocellulose membranes (Cat No: 162-0112), from BioRad (Hercules, CA); and Pierce ECL western blot substrate from Fisher Scientific (Rockford, IL, Cat No: 34075); and protease inhibitor cocktail from Roche (Indianapolis, IN, Cat No: 04-693-124-001).

Antibodies utilized in this study include Rabbit anti-STAT3 (pSer727, Cat No: 9134), STAT3 (Cat No: 9132), Akt (pSer473, Cat No: 4721), Akt (Cat No: 9272), AMPk (pThr172, Cat No: 2531), and AMPk (Cat No: 2532) purchased from Cell Signaling (Danvers, MA). Mouse anti-β-Actin (Cat No: 5441) antibody was purchased from Sigma, and Sheep anti-Mouse IgG (Cat No: NA931V) and Donkey anti-Rabbit IgG (Cat No: NA934V) were purchased from GE healthcare, Piscataway, NJ.

### Cell Culture and Differentiation

SH-SY5Y cells were maintained in DMEM/F12 (1∶1) media, supplemented with 10% (v/v) Fetal Bovine Serum and 1% (v/v) Pen/Strep. SH-SY5Y cells were differentiated in DMEM/F12 media containing 10 µM retinoic acid for 7 days and given fresh DMEM/F12 media with 10 µM retinoic acid every 48 hours.

Preadipocytes (3T3-L1 cells) were maintained in high glucose-DMEM supplemented with 10% (v/v) Calf Serum and 1% (v/v) pen/strep. 3T3-L1 cells were differentiated for three days in differentiation media DMEM with 10% (v/v) FBS, containing 1 µg/ml insulin, 1 µM Dexamethasone, and 0.5 mM IBMX and further matured for four days in DMEM media containing 10%(v/v) FBS and 1 µg/ml insulin.

Cells were maintained in 25 cm2 flasks at 37°C and 7% CO2 to approximately 95% confluency and subsequently passaged with 0.05% Trypsin/EDTA.

### Knockdown of FTO Expression Utilizing Small-Interfering RNAs

3T3-L1 and SH-SY5Y cells were transfected with either anti-FTO-siRNA purchased from Dharmacon (Lafayette, CO, Cat No: D-004159-02) or Signal Silence Control siRNA (Cat No: 6568S, Cell Signaling Technology), that does not lead to the specific degradation of any cellular message, using either DharmaFECT 3 (3T3-L1) or DharmaFECT 2 (SH-SY5Y) transfection reagent, respectively, according to the manufacturers specifications. Briefly, cells were plated at appropriate cell concentrations (indicated in specific experiments), and after 24 hours, plated cells were rinsed 2× with Opti-MEM media and pre-incubated for 30 minutes in Opti-MEM media at 37°C and 7% CO_2._ A complex of 100 nM siRNA and DharmaFECT transfection reagent in Opti-MEM was then added, and cells were incubated for 24 hours in transfection media. After 24 hours, the transfection media was removed, cells were washed 1× and incubated for 24 hours with the appropriate media as described earlier. After a total of 48 hours from the start of transfection, naïve cells were used for experimentation or differentiation. 48 hours was selected since maximum downregulation of FTO occurred at this point. To achieve stable transfection of siRNA during cellular differentiation, cells were transfected for 48 hours as described above and subsequently exposed to the differentiating agents as described in the previous section. qRT-PCR was used to confirm FTO knockdown using Anti-GAPD siRNA (Dharmacon D-001140-01) as a positive control.

### qRT-PCR

To analyze mRNA expression of FTO, NPY and POMC; 3T3-L1 and SH-SY5Y cells were plated at 2×10^4^ or 3×10^5^ respectively in 35 mm petri dishes and transfected with siRNA as described previously. After 24 hours, dishes were washed 1× with media and replaced with appropriate media. After an additional 24 hours (48 hours after the start of transfection) or after differentiation, cells were rinsed 2× with ice-cold PBS and total RNA was isolated from siRNA treated cells using the PureLink Micro-to-Midi™ Total RNA Purification System from Invitrogen (Cat No: 12183-018). cDNA synthesis and qPCR was performed using the Two-Step qRT-PCR Kit from Invitrogen (Cat No: 11748-100), as prescribed by the manufacturer and using the Applied Biosciences 7300 Real-Time PCR System. Primers used were obtained from Integrated DNA Technologies (Coralville, Iowa) and the sequence was as follows: FTO forward: 5′ GAA TTC TAT CAG CAG TGG CAG CTG 3′, FTO reverse; 5′ AGC CAT GCT TGT GCA GTG TG 3′, GAPDH forward: 5′ TTG CCA ATG ACC CCT TCA 3′, GAPD reverse: 5′ CGC CCC ACT TGA TTT TGG A 3′, NPY forward: 5′ TCC AGC CCA GAG ACA CTG ATT 3′, NPY reverse: 5′ AGG GTC TTC AAG CCG AGT TCT3′, POMC forward: 5′ AGC CCG CCC AAG GAC AAG 3′, POMC reverse: 5′ TGC CCT CAC TCG CCC TTC T 3′.

### Immunoblotting

To determine the effect of FTO knockdown on expression levels of involved proteins, SH-SY5Y and 3T3-L1 cells were plated at 3×10^5^ or 2×10^4^ cells per 35 mm petri dish, respectively, and transfected with siRNA as described previously. After 24 hours, dishes were washed 1× with media and replaced with appropriate media. After an additional 24 hours (48 total hours of transfection) or after differentiation, cells were rinsed 2× with ice-cold PBS, and lysed in phospho-protein extraction buffer with protease inhibitor cocktail (Roche) as described previously [40]. Cell lysates were separated by 7.5% or 10% SDS-PAGE under reducing conditions. Proteins were transferred to a nitrocellulose immobilization membrane and immunoblotted using the enhanced ECL.

### Determination of Cell Viability

To determine the effect of oligomycin and FTO siRNA on the survival of 3T3-L1 adipocytes and SH-SY5Y neurons, cells were plated at 50,000 cells or 200,000 cells per well, respectively, in 12 well plates in DMEM and appropriate media, as described above. After 24 hours, cells were transfected with either FTO or Control siRNA as described above for 48 hours. Cells were then treated in the presence and absence of 10 mM oligomycin for 30 minutes after which cells were trypsinized and counted with trypan blue exclusion. Each data point was repeated in quadruplicate.

### ATP Assay

To determine if changes in FTO expression modulate energy balance in SH-SY5Y and 3T3-L1 cells, we quantified the levels of ATP present within cells with or without FTO downregulation. ATP content of cells was measured using the Perkin Elmer ATPlite luminescence assay system (Waltham, MA, Cat No: 6016941). Briefly, SH-SY5Y and 3T3-L1 cells were plated at 2×10^3^ and 3×10^3^ cells/well, respectively, in a 96 well plate, and transfected as described previously. After 24 hours, wells were washed 1× with media and replaced with appropriate media. After an additional 24 hours (48 total hours of transfection) or after differentiation, cells were rinsed 2× with ice-cold PBS and incubated for 30 minutes in glucose-free media with or without 10 mM oligomycin, a specific inhibitor of ATP synthase to reduce levels of endogenous ATP. Cells were then lysed with the provided 1× mammalian cell lysis buffer for 5 minutes on an orbital shaker. After which the provided reconstituted luciferase substrate was then added to the cell lysate and incubated for 5 minutes. The plates were then incubated in dark for 10 minutes, and luminescence was measured using the Bio Tek Synergy 2 multi-mode microplate reader (Winooski, TN).

### Glucose Uptake Assay

2-[N-(7-nitrobenz-2-oxa-1,3-diazol-4-yl) amino]-2-deoxy-d-glucose (2-NBDG) (Invitrogen, Cat No: N13195), a non-hydrolysable fluorescent glucose analog with a excitation/emission maxima of ∼465/540 nm [41], was used to measure basal uptake of glucose into SH-SY5Y cells. Briefly, SH-SY5Y and 3T3-L1 cells were plated at 3×103 and 2×103 cells/well, respectively, in a 96 well plate, and transfected as described previously. After 24 hours, wells were washed 1× in media and replaced with appropriate media. After another 24 hours (48 total hours of transfection) or after differentiation, cells were washed 1× with DMEM media lacking glucose and/or sodium pyruvate (Invitrogen, Cat No: 11966-025) and exposed to 80 µM 2-NBDG for 5 minutes in glucose-free DMEM media. 2-NBDG containing glucose-free DMEM was then removed and cells were washed 2× with ice-cold HBSS with Ca2+ and Mg2+ (Invitrogen, Cat No: 14025-092) to remove residual 2-NBDG. Cell-associated fluorescent signal was analyzed using the Bio Tek Synergy 2 multi-mode microplate reader.

### Analysis of Results

qPCR results were analyzed by the Applied Biosciences 7300 Real-Time PCR System software with GAPD mRNA serving as a positive internal control. Immunoblotting results were analyzed by densitometric analysis using the Image J software from the National Institutes of Health with values representing percent of control from an average of triplicate experiments. Experiments on cell viability were analyzed using repeated measures ANOVA with α = 0.05. ATP and Glucose experiments were performed in with paired control groups in “N” number of replicates as indicated, respectively, and analyzed against the appropriate control siRNA and diluent groups using a one-way ANOVA with post-hoc analysis. Data is presented as FTO siRNA vs Control siRNA, because diluent groups were found be uniformly similar to Control siRNA groups. Significance was established at α = 0.05.

## References

[1] Dina C, Meyre D, Gallina S, Durand E, Korner A (2007). Variation in FTO contributes to childhood obesity and severe adult obesity.. Nat Genet.

[2] Frayling TM, Timpson NJ, Weedon MN, Zeggini E, Freathy RM (2007). A common variant in the FTO gene is associated with body mass index and predisposes to childhood and adult obesity.. Science.

[3] Wardle J, Carnell S, Haworth CM, Farooqi IS, O’Rahilly S (2008). Obesity associated genetic variation in FTO is associated with diminished satiety.. J Clin Endocrinol Metab.

[4] Speakman JR, Rance KA, Johnstone AM (2008). Polymorphisms of the FTO gene are associated with variation in energy intake, but not energy expenditure.. Obesity (Silver Spring).

[5] Church C, Moir L, McMurray F, Girard C, Banks GT (2010). Overexpression of Fto leads to increased food intake and results in obesity.. Nat Genet.

[6] Church C, Lee S, Bagg EA, McTaggart JS, Deacon R (2009). A mouse model for the metabolic effects of the human fat mass and obesity associated FTO gene.. PLoS Genet.

[7] Fischer J, Koch L, Emmerling C, Vierkotten J, Peters T (2009). Inactivation of the Fto gene protects from obesity.. Nature.

[8] Fredriksson R, Hagglund M, Olszewski PK, Stephansson O, Jacobsson JA (2008). The obesity gene, FTO, is of ancient origin, up-regulated during food deprivation and expressed in neurons of feeding-related nuclei of the brain.. Endocrinology.

[9] Poritsanos NJ, Lew PS, Mizuno TM (2010). Relationship between blood glucose levels and hepatic Fto mRNA expression in mice.. Biochem Biophys Res Commun.

[10] Wahlen K, Sjolin E, Hoffstedt J (2008). The common rs9939609 gene variant of the fat mass- and obesity-associated gene FTO is related to fat cell lipolysis.. J Lipid Res.

[11] Olszewski PK, Fredriksson R, Olszewska AM, Stephansson O, Alsio J (2009). Hypothalamic FTO is associated with the regulation of energy intake not feeding reward.. BMC Neurosci.

[12] Grunnet LG, Nilsson E, Ling C, Hansen T, Pedersen O (2009). Regulation and function of FTO mRNA expression in human skeletal muscle and subcutaneous adipose tissue.. Diabetes.

[13] Ropelle ER, Fernandes MF, Flores MB, Ueno M, Rocco S (2008). Central exercise action increases the AMPK and mTOR response to leptin.. PLoS One.

[14] Minokoshi Y, Alquier T, Furukawa N, Kim YB, Lee A (2004). AMP-kinase regulates food intake by responding to hormonal and nutrient signals in the hypothalamus.. Nature.

[15] Jing M, Ismail-Beigi F (2006). Role of 5'-AMP-activated protein kinase in stimulation of glucose transport in response to inhibition of oxidative phosphorylation.. Am J Physiol Cell Physiol.

[16] Kola B (2008). Role of AMP-activated protein kinase in the control of appetite.. J Neuroendocrinol.

[17] Shaw RJ, Kosmatka M, Bardeesy N, Hurley RL, Witters LA (2004). The tumor suppressor LKB1 kinase directly activates AMP-activated kinase and regulates apoptosis in response to energy stress.. Proc Natl Acad Sci U S A.

[18] Bae SS, Cho H, Mu J, Birnbaum MJ (2003). Isoform-specific regulation of insulin-dependent glucose uptake by Akt/protein kinase B. J Biol Chem.

[19] Cecil JE, Tavendale R, Watt P, Hetherington MM, Palmer CN (2008). An obesity-associated FTO gene variant and increased energy intake in children.. N Engl J Med.

[20] Rutters F, Lemmens SG, Born JM, Bouwman F, Nieuwenhuizen AG (2010). Genetic associations with acute stress-related changes in eating in the absence of hunger.. Patient Educ Couns.

[21] Haupt A, Thamer C, Staiger H, Tschritter O, Kirchhoff K (2009). Variation in the FTO gene influences food intake but not energy expenditure.. Exp Clin Endocrinol Diabetes.

[22] Bates SH, Stearns WH, Dundon TA, Schubert M, Tso AW (2003). STAT3 signalling is required for leptin regulation of energy balance but not reproduction.. Nature.

[23] Coll AP, Loraine Tung YC (2009). Pro-opiomelanocortin (POMC)-derived peptides and the regulation of energy homeostasis.. Mol Cell Endocrinol.

[24] Gerken T, Girard CA, Tung YC, Webby CJ, Saudek V (2007). The obesity-associated FTO gene encodes a 2-oxoglutarate-dependent nucleic acid demethylase.. Science.

[25] Wu Q, Saunders RA, Szkudlarek-Mikho M, Serna Ide L, Chin KV (2010). The obesity-associated Fto gene is a transcriptional coactivator.. Biochem Biophys Res Commun.

[26] Bravard A, Lefai E, Meugnier E, Pesenti S, Disse E (2011). FTO is increased in muscle during type 2 diabetes, and its overexpression in myotubes alters insulin signaling, enhances lipogenesis and ROS production, and induces mitochondrial dysfunction.. Diabetes.

[27] Grunnet LG, Brons C, Jacobsen S, Nilsson E, Astrup A (2009). Increased recovery rates of phosphocreatine and inorganic phosphate after isometric contraction in oxidative muscle fibers and elevated hepatic insulin resistance in homozygous carriers of the A-allele of FTO rs9939609.. J Clin Endocrinol Metab.

[28] Sakoda H, Ogihara T, Anai M, Fujishiro M, Ono H (2002). Activation of AMPK is essential for AICAR-induced glucose uptake by skeletal muscle but not adipocytes.. Am J Physiol Endocrinol Metab.

[29] Shah AK, Gupta A, Dey CS (2011). AICAR induced AMPK activation potentiates neuronal insulin signaling and glucose uptake.. Arch Biochem Biophys.

[30] Russo VC, Kobayashi K, Najdovska S, Baker NL, Werther GA (2004). Neuronal protection from glucose deprivation via modulation of glucose transport and inhibition of apoptosis: a role for the insulin-like growth factor system.. Brain Res.

[31] Blodgett DM, De Zutter JK, Levine KB, Karim P, Carruthers A (2007). Structural basis of GLUT1 inhibition by cytoplasmic ATP.. J Gen Physiol.

[32] Gonzalez JA, Reimann F, Burdakov D (2009). Dissociation between sensing and metabolism of glucose in sugar sensing neurones.. J Physiol.

[33] Jequier E (2002). Leptin signaling, adiposity, and energy balance.. Ann N Y Acad Sci.

[34] Kohno D, Sone H, Tanaka S, Kurita H, Gantulga D (2011). AMP-activated protein kinase activates neuropeptide Y neurons in the hypothalamic arcuate nucleus to increase food intake in rats.. Neurosci Lett.

[35] Tung YC, Ayuso E, Shan X, Bosch F, O’Rahilly S (2010). Hypothalamic-specific manipulation of Fto, the ortholog of the human obesity gene FTO, affects food intake in rats.. PLoS One.

[36] Berulava T, Horsthemke B (2010). The obesity-associated SNPs in intron 1 of the FTO gene affect primary transcript levels.. Eur J Hum Genet.

[37] Pahlman S, Ruusala AI, Abrahamsson L, Mattsson ME, Esscher T (1984). Retinoic acid-induced differentiation of cultured human neuroblastoma cells: a comparison with phorbolester-induced differentiation.. Cell Differ.

[38] Sadowski HB, Wheeler TT, Young DA (1992). Gene expression during 3T3-L1 adipocyte differentiation. Characterization of initial responses to the inducing agents and changes during commitment to differentiation.. J Biol Chem.

[39] Tews D, Fischer-Posovszky P, Wabitsch M (2010). Regulation of FTO and FTM expression during human preadipocyte differentiation.. Horm Metab Res.

[40] Puri N, Ahmed S, Janamanchi V, Tretiakova M, Zumba O (2007). c-Met is a potentially new therapeutic target for treatment of human melanoma.. Clin Cancer Res.

[41] Zou C, Wang Y, Shen Z (2005). 2-NBDG as a fluorescent indicator for direct glucose uptake measurement.. J Biochem Biophys Methods.

